# Abstracts/Sommaires/Zusammenfassungen

**DOI:** 10.1155/S1463924680000533

**Published:** 1980


### Abstracts Sommaires Zusammenfassungen

### Page 183

**Improved accuracy in automated chemistry through the use of reference materials**
R.F. Coleman

The development of automated analyses has led to poor accuracy in some cases in order to keep costs low and throughput high. It is argued that accuracy can be improved by using methods and reference materials which can be traced back to a national standards laboratory.

**Exactitude améliorée en chimie automatisée par l'emploi de matériaux de référence**

Le développement d'analyses automatisées à conduit – dans certains cas – à une faible précision, dans le but d'atteindre une haute capacité à peu de frais. On prêtend que la prêcision peut être améliorée par l'emploi de méthodes et matériaux de référence qui peuvent être retracês vers un laboratoire national de standards.

**Verbesserte Genauigkeit bei der automatisierten chemischen Analytik durch Benützung von Referenzmaterialien**

Die Entwicklung automatisierter Analysen führte in einigen Fällen wegen des Bestrebens zu Kostensenkung und hohem Durchsatz zu schlechter Genauigkeit. Es wird nun vorgetragen, dass die Genauigkeit durch Benützung von Methoden und Referenzmaterialien, welche auf ein nationales Eich- und Prüflaboratorium zurückgefuhrt werden können, verbessert werden kann.

### Page 186

**Simple method for calculating frequency of standardisation**
J. Inczedy

A simple method for calculation of optimal standardisation time interval in analytical measurements is described. The method is based on the assumptions that the motion of the drift is periodic and can be approximated by a sine wave function. Using the amplitude and period time of the function and the standard deviation of the short time analytical measurements, the optimal time interval between standardisations can be estimated.

**Méthode simple pour la calculation de la fréquence de standardisation**

Une méthode simple pour la calculation de la standardisation optimale d'un intervalle de temps dan le mesurage analytique est décrite. La méthode est basée sur la supposition que le mouvement de la dérive est périodique et peut être rapproché par une fonction sinuosidale. En employant l'amplitude et la période de fonction et la déviation standard de séries de mesures analytiques, l'intervalle optimal entre les standardisations peut être estimé.

**Einfache Methode zur Bestimmung der Standardisierungsfrequenz**

Eine einfache Methode zur Berechnung des optimalen Zeitintervalls zwischen Standardisierungen in analytischen Instrumenten wird beschrieben. Die Methode basiert auf der Annahme, dass die Driftbewegung periodisch ist und durch eine Sinuskurve angenähert werden kann. Unter Benützung von Amplitude und Periodenzeit der Funktion sowie der kurzfristigen Standardabweichungen der analytischen Messungen kann das optimale Zeitintervall zwischen Standardisierung abgeschätzt werden.

### Page 189

**Amperometric measurement system for the determination of oxidase enzyme dependent reactions by continuous flow and flow injection analysis**
E. L. Gulberg, A. S. Attiyat, and G. D. Christian

Electrochemical detection systems for oxidase enzyme dependent reactions based upon the Mo(VI) catalysed oxidation of iodide by hydrogen peroxide are studied. The iodine produced is monitored amperometrically using a flow cell. Continuous flow analysis and flow injection analysis were both studied. A glass column containing immobilised alcohol oxidase was used in the continuous flow determination of ethanol.

**Système de mesure ampérométrique pour la détermination de réactions dépendant de l' enzyme oxidase par analyse à débit continu et par "flow injection"**

Des systèmes de détection electrochimique pour des réactions dépendant de l'enzyme oxidase basé sur l'oxidation du iodure par l'eau oxigénée catalysée par le Mo(VI) sont étudiés. Le iode produit est contrôlé par ampérométrie dans une cellule à débit continu. L'analyse par débit continu et par "flow injection" ont été étudiées. Une colonne en ver contenant de l'oxidase d' alcool immobilisée a été utilisée pour la determination de l'éthanol par débit continu.

**Amperometrisches Mess-System für die Bestimmung von Oxidase-Enzym abhängigen Reaktionen mittels "Continuous Flow" und "Flow Injection Analysis"**

Die vorliegende Arbeit ist eine Studie über elektrochemische Detektionssysteme für von Enzyme Oxidase abhängige Reaktionen, die auf der durch Mo(VI) katalysierten Oxidation von Iod durch Wasserstoffperoxyd basieren. Das produzierte Iod wird in einer Durchflusszelle amperometrisch überwacht. Kontinuierliche Durchflussanalyse und Einspritz-Durchflussanalyse werden studiert. Für die kontinuierliche Durchflussbestimmung von Aethanol wurde ein Glaskolonne mit immobilisierter Alkohol-Oxidase verwendet.

### Page 194

**Interfacing a titrator to a microcomputer for incremental or continuous modes of operation**
M. Danczíger and H. V. Malmstadt

Specific interface circuits and instrumental methodology are presented to show how manual or semi-automatic titrators can be automated and made more interactive and versatile by connecting to a microcomputer. The rather tedious, but often advantageous, incremental endpoint techniques and calculation procedures of Kolthoff, Fortuin, Wolf, Keller-Richter, and Bartscher, as well as the continuous titrant delivery mode, are readily implemented with this system. Data are presented to provide comparisons between the various incremental techniques for both a weak acid-strong base and a precipitation titration. In most cases, the precisions for the titrations are about 0.1% RSD, similar to the reproducibility for the sample-handling devices used.

**Interface d'un titrateur avec microordinateur pour titrages par incréments ou en mode continu**

Des circuits d'interface speciaux ainsi que la méthodologie instrumentale sont présenté afin de montrer comment des titrateurs manuels ou semiautomatiques peuvent être automatisée et rendus plus interactifs et versatiles en les couplant à un microordinatuer. Les méthodes incrémentielles souvent nécessaries bien que ennuyeuses avec les calculs selon Kolthoff, Fortuin, Wolf, Keller-Richter et Bartscher ainsi que le mode de titrage en continu sont faciles à implémenter avec ce système. Des données sont présentées pour permettre la comparaison entre les différents modes incrémentiels pour les titrage d'acides faibles par bases fortes et pour les titrages de précipitation. Dans la plupart des cas la précision était très bonne donnant un écart-type de 0.1% similaire à la reproductibilité de la manipulation des échantillons.

**Mikrocomputersteuerung zu einem Titrator für inkrementelle oder kontinuierliche Betriebsart**

Es werden spezifische Interface-Schaltungen und die instrumentelle Methodologie dargelegt um zu zeigen, wie manuelle oder halbautomatische Titratoren durch Anschluss an einen Mikrocomputer automatisiert und interaktiver und vielseitiger verwendbar gemacht werden können. Die eher mühsamen aber oft vorteilhaften inkrementellen Endpunktmethoden und Berechnungen von Kolthoff, Fortuin, Wolf, Keller-Richter und Bartscher als auch die Methode der kontinuierlichen Zugabe des Titrationsmittels können auf diesem System leicht realisiert werden. Es werden Resultate vorgelegt, die einen Vergleich zwischen den verschiedenen inkrementellen Techniken ermöglichen für die Titration schwacher Säuren mit starken Basen sowie für Fällungs-Titrationen. In den meisten Fällen ergibt sich eine Präzision der Titrationen von 0.1% RSD, was nahe bei der Reproduzierbarkeit der verwendeten Geräte für die Probenvorbereitung liegt.

### Page 200

**The flexible dialyser membrane – a source of error in plasma creatinine determination**

T.A. Walmsley, R.T. Fowler, M.H. Abernethy, M. Lever and D.J. Munster

The dialyser membrane in a continuous flow analyser stretches and relaxes as the pressure difference across the membrane changes during an analysis. The greatest conformational change occurs when the pressure difference across the membrane is zero because of slackness of the dialyser membrane. The changes in conformation of the dialyser membrane momentarily affect the flow rate of the recipient stream from the dialyser and change the dilution of any reagent added after dialysis.

**La membrane de dialyse flexible – source d'erreurs dans la détermination de la créatinine de plasma**

La membrane de dialyse dans un analysateur à flux continu se tend et se détend quand la différence de pression change à travers la membrane pendant l'analyse. Le plus grand changement conforme se présente quand la différence de pression à travers la membrane est zéro à cause du relâchement de la membrane de dialyse. Les changements de conformité de la membrane de dialyse affectent momentanément la proportion du flux du courant de récipient venant du dialyseur et change la dilution de tout réagent ajouté après la dialyse.

**Die flexible Dialysator-Membran – Fehlerquelle bei der Plasma-Kreatininbestimmung**

Die Dialysator-Membran in kontinuierlichen Analysatoren dehnt sich und zieht sich wieder zusammen als Folge der Druckschwankungen an der Membrane während der Analyse. Die grösste Formänderung entsteht dabei, wenn der Druckabfall an der Membran null ist, weil die Dialysator-Membran dann lose ist. Die Formänderungen in der Dialysator-Membran beeinflussen kurzfristig die Strömungsgeschwindigkeit auf der Rückseite des Dialysators und verändern damit die Verdünnung eines nach der Dialyse zugegebenen Reagens.

### Page 202

**Bichromatic method for the enzymatic determination of serum triglycerides with the GEMSAEC Fast analyser**

P. Bijster, H. L. Vader and C. L. J. Vink

A bichromatic enzymatic analysis is described for serum triglycerides using a GEMSAEC fast analyser. It is based on an equilibrium measurement without extrapolation and takes only two minutes instrument time. To minimise optical interferences, the absorbance of the reaction mixture is read after extra-instrumental hydrolysis of triglycerides and elimination of glycerol, at two wavelengths (340 nm and 380 nm). This method is precise and accurate, although the interference caused by haemoglobin and bilirubin are not completely minimised.

**Une méthode bichromatique pour la détermination enzymatique de triglycérides dans le serum avec l'analyseur rapide GEMSAEC**

Une analyse enzymatique bichromatique des triglycérides dans le serum utilisant un analyseur rapide GEMSAEC est décrite. Elle est basée sur une mesure d'équilibre sans extrapolation et occupe l'instrument pendant deux minutes seulement. Afin de minimaliser les interférences optiques l'absorption du mélange de réaction est mesuré, apres l' hydrolyse externe et l'élimination des triglycérides, à deux longeuers d'onde (340 nm et 380 nm). Cette méthode est précise et exacte, bien que les interférences causées par l'hémoglobin et le bilirubin n'ont pas pu être complètement minimalisées.

**Eine bichromatische Methode für die enzymatische Bestimmung von Triglyzerid in Serum mit dem GEMSAEC Fast Analyzer**

In der vorliegenden Arbeit wird eine bichromatische Enzym-Analyse für Triglyzerid in Serum mit dem GEMSAEC Fas Analyzer beschrieben. Sie basiert auf einer Gleichgewichtsmessung ohne Extrapolation und beansprucht auf dem Instrument nur 2 Minuten. Zur Minimalisierung optischer Interferenzen wird die Absorption der Reaktionslösung bei zwei Wellenlängen (340 nm und 380 nm) abgelesen, nachdem ausserhalb des Instruments die Hydrolyse der Triglyzeride und die Elimination von Glyzerin durchgeführt wurde. Die Methode ist präzis und genau obwohl die Interferenze durch Hämoglobin und Bilirubin nicht vollständig minimalisiert sind.

### Page 206

**A clinical appraisal of the Greiner G300 clinical chemistry analyser**

M. J. Hallworth, S. G. Archer and J. G. Lines

A short evaluation of the Greiner G300 analyser in routine clincial chemistry use – that is, analysis of patient samples – is reported. Data on precision (within-run and day-to-day) and carryover are presented, and the accuracy of the instrument is assessed by comparison with continuous-flow methods and by analysis of quality control materials. The possible contribution of the G300 towards handling the workload of clinical chemistry laboratories of varying size is discussed, and in particular its use as a single instrument to encompass both adult emergency and routine paediatric work in large laboratories is emphasised.

**Etude clinique de l'analyseur Greiner G300 pour chimie clinique**

Une briève évaluation de l'analyseur Greiner G300 dans la routine de la chimie clinique – c'est à dire l'analyse d'échantillons de malades – est décrite. Des données sur la précision (dans une série et de jour-en-jour) et la contamination sont présentées, l'exactitude de l'instrument est vérifiée par comparaison avec des méthodes à débit continu et par l'analyse de matériel pour contrôle de qualité. La capabilité due G300 de remplir les nécessités de laboratoires de chimie clinique de tailles différentes est discutée. En particulier l'utilisation d'un seul instrument pour le traitement des cas d'urgance d'adultes et pour la routine pédiatrique dans des grands laboratoires est soulignée.

**Klinische Beurteilung des GREINER G300 Clinical Chemistry Analyzer**

Es wird über eine kurze Evaluation des Greiner G300 Analysators im Routinebetrieb der klinischen Chemie – d.h. Analyse von Patienten-Proben – berichtet. Daten zur Präzision (innerhalb einer Serie und von Tag zu Tag) und zur Verschleppung werden vorgelegt, und die Genauigkeit des Instruments wird beurteilt im Vergleich zu den kontinuierlichen Durchflussmethoden und durch Analyse von Materialien zur Qualitätskontrolle. Der mögliche Beitrag, den ein G300 zuhanden des Arbeitsanfalls in klinisch-chemischen Laboratorien verschiedenster Grössen leisten kann, wird diskutiert. Speziell betont wird die Verwendung als Einzelinstrument zur Bewältigung von Notfallproben Erwachsener und von pädiatrischer Routine-Arbeit in grossen Laboratorien.

### Page 212

**An evaluation of the NOVA 1 ion selective electrode analyser for sodium and potassium determination**

W. Annan, N. A. Kirwan, W. S. Robertson and P. R. Teasdale

The paper describes a laboratory evaluation of a NOVA 1 ion-selective electrode analyser for the determination of sodium and potassium by direct potentiometry in undiluted whole blood, plasma or serum and in urine after dilution. The instrument was considered to be precise with an average between batch coefficients of variation of 0.5% and 1.4% respectively for sodium and potassium in plasma and 2.1% and 2.7% in urine.

**Evaluation de l'analyseur NOVA 1 avec electrodes selectives pour soude et potasse**

Nous decrivons une évaluation en laboratoire de l'analisuer NOVA 1 pour la détermination de soude et potasse par potentiométrie directe avec électrodes sélectives dans le sang (non dilué), le plasma, le serum et dans l' urine après dilution. La précision de l' instrument est considérée bonne avec des coéfficients de variation de 0.5% et 1.4% pour la soude et la potasse dans le plasma et de 2.1% resp. 2.7% dans l'urine.

**Evaluation des NOVA 1 Ion Selective Electrode Analyzer für die Bestimmung von Natrium und Kalium**

Diese Arbeit beschreibt eine Evaluation für das Labor des NOVA 1 Ion Selective Electrode Analyzer für die Bestimmung von Natrium und Kalium mit Direktpotentiometrie in unverdünntem Blut, Plasma oder Serum und nach Verdünnung in Urin. Die beohachtete Präzision ergab einen mittleren Variationskoeffizient der Serien von 0.5% für Natrium und 1.4% für Kalium in Plasma beziehungsweise 2.1% und 2.7% in Urin.

